# Inter-departmental abortion travels in metropolitan France: A mixed-methods analysis of women’s experiences, access, and barriers to abortion care

**DOI:** 10.1371/journal.pone.0273190

**Published:** 2022-10-04

**Authors:** Laura Rahm, Silvia De Zordo, Joanna Mishtal, Camille L. Garnsey, Caitlin Gerdts

**Affiliations:** 1 Department of Anthropology, University of Barcelona, Barcelona, Spain; 2 Department of Anthropology, University of Central Florida, Orlando, FL, United States of America; 3 Independent Research Consultant & Fulbright US Student, Storrs, CT, United States of America; 4 Ibis Reproductive Health, Oakland, CA, United States of America; University of Salamanca, SPAIN

## Abstract

In Europe, there is a dearth of studies on abortion-related mobilities within countries where abortion is legal. In France, 18% of women seek abortion care outside their department of residence care. Most of these flows take place within Île-de-France region. This paper aims at providing novel insights into the motives and experiences of women traveling within France and particularly within the Île-de-France region for abortion care. It draws upon official abortion statistics as well as quantitative and qualitative data collected in three Parisian hospitals during a five-year European research project on barriers to legal abortion and abortion travel. Despite governmental efforts to facilitate access to abortions over the past decades, our findings show that various barriers exist for why women do not find services in their department of residence (lack of services or access to preferred methods, quality of care, long waiting times). However, most of our study participants report coming to Paris as a convenience and use commuting as a strategy to overcome obstacles in receiving abortion care.

## 1. Introduction

While much research has been published about women traveling for abortions in contexts where abortion laws are restrictive [1–4], less is known about women’s travel for care in countries with relatively liberal abortion laws [5]. Europe makes for an interesting case. Abortions are legal upon request, or on broad social or economic grounds, in nearly all-European countries. However, women exceeding the gestational age limit of their country of origin frequently need to travel abroad for an abortion [5].

Another facet of abortion-related travel concerns the in-country mobility of women who cannot find services in their department of residence. France is a case study of inter-departmental mobility issues. The French government has made great efforts to liberalize the abortion law and facilitate access over the past decades [6–8], particularly by authorizing medical abortion outside the hospital (in 2004), which led to a decrease in the number of surgical abortions (only very recently surgical abortion has started to be performed outside the hospital). The gestational age limit was increased from 10 to 12 weeks gestation in 2001 and recently to 14 weeks, after a long political debate [9, 10]. General practitioners can provide abortion care since 2004 and midwifes since 2016. The French social insurance system covers 100% of abortion costs disregarding of the provider (public or private). These legal and organizational changes have had an impact on the provision of abortion care and of specific methods, as recent national and regional data on abortion provision show.

In 2019, 217,536 abortions were recorded in metropolitan France. The data were obtained from DREES [11] unless otherwise indicated. Table 1 shows the abortions performed in metropolitan France, by women’s region of residence, place of abortion and method, in 2019.

**Table 1:** Number of abortions by region of residence of the woman, place of abortion and method, metropolitan France, 2019.

	2019						
	Abortion in hospitals			Out-of-hospital abortion		TOTAL IVG	Abortion rate (per 1000 women aged 15–49)
Region	Surgical	Medical	method not specified	private practice	centers*		
Auvergne- Rhône-Alpes	8202	9395	146	5473	715	23931	13,9
Bourgogne-Franche-Comté	2206	2864	401	1741	103	7315	13,3
Bretagne	3074	4061	25	963	112	8235	12,3
Centre-Val de Loire	2186	3628	35	1149	130	7128	13,9
Corse	242	874	71	180	6	1373	19,4
Grand Est	3710	9789	113	1279	121	15012	12,9
Hauts-de-France	5698	9725	95	2686	379	18583	14,2
Île-de-France	16773	18321	328	17169	2672	55263	18,5
Normandie	2355	4751	60	1632	121	8919	13,2
Nouvelle-Aquitaine	5438	7227	63	3870	762	17360	14,5
Occitanie	6058	9451	116	5500	296	21421	17,6
Pays de la Loire	4224	4523	40	526	18	9331	11,8
Provence-Alpes-Côte d’Azur	6173	9525	233	7423	311	23665	22,9
**Total metropolitan France**	**66339**	**94134**	**1726**	**49591**	**5746**	**217536**	**15,6**

* Abortions performed in health centers and family planning centers (Centre de Planification et d’Éducation Familiale, CPEFs)

Source: [11]

In 2019, a total 162,199 abortions (75%) were performed inside the hospital sector: 94,134 (58%) were medical, 66,339 (41%) surgical, and 1726 (1%) were not specified methods. Regional differences are noticeable. The usage of surgical abortions varies across regions, from 20% in Corse to 48% in Pays de la Loire (Table 1).

One quarter (25%) of all abortions in metropolitan France were performed outside the hospital sector in 2019, mainly in private practices (49,591) and to a lesser extent in health centers or family planning centers, CPEFs (5,746). Important regional variations emerge: less than 10% of abortions are performed in private practices in Pays de la Loire and Grand-Est regions in 2019, whereas 31% in Provence-Alpes-Côte d’Azur and Île-de-France. The performance of abortions in health centers and CPEFs is gradually developing, reaching 4% (762 abortions) in Nouvelle-Aquitaine, and 5% (2,672 abortions) in Île-de-France (Table 1).

In 2019, 70% of abortions were performed medically, compared to 30% in 2001. Meanwhile, the number of surgical abortions has decreased, particularly in private health facilities, from 24% of all abortions in 2001 to 4% in 2019 [12].

The abortion rate in metropolitan France was 15.6 abortions per 1,000 women aged 15 to 49 in 2019 with significant regional variations, ranging from 11.8 per 1,000 women in Pays de la Loire to 22.9 in Provence-Alpes-Côte d’Azur. In terms of absolute numbers, most abortions are concentrated in the Île-de-France region (55,263), followed by the regions Auvergne-Rhône-Alpes (23,931) and Provence-Alpes-Côte d’Azur (23,665) (Table 1). Most abortion related mobilities are also concentrated in the Île-de-France region, where we carried out data collection.

Inter-departmental abortion travel refers to in-country flows of women seeking abortion care outside their department of residence. This can be in neighboring departments within their own region of residence, or in other regions. We focus specifically on our data from Île de France. We had planned to do data collection in other regions, but the pandemic obliged us to stop data collection for a long period of time and to revise our original research plans.

Despite legal and organizational changes made to improve access to abortion care in France, including medical abortion via teleconsultation, which was authorized and promoted during the COVID-19 pandemic [13–15], barriers to access persist. Some of them lead women to seek abortion care outside their department of residence.

Several barriers have already been identified through governmental and non-governmental reports that highlight regional differences in access to care: declining numbers of (private) abortion services, conscientious objection, and difficulties in accessing second trimester abortion for psychosocial reasons [16–19]. However, not much is known about the origin and destination areas of inter-departmental abortion travels in France and the experiences of women undertaking such trips.

## 2. Objective

This paper aims at documenting and mapping abortion travels in metropolitan France. We explore how frequently women travel in France, and from where/to where they travel to access abortion care within France. Secondly, it seeks to explore pregnant women’s experiences with travel for abortion care within France, specifically those commuting within Île de France. We document the socio-demographic characteristics and reproductive histories of our study participants traveling for abortion care within this region—compared with abortion seekers who do not travel (whom we labeled as “residents”). We also explore the primary reasons why they seek abortion care in other health departments and examine the associated costs with inter-departmental abortion travels. Our initial hypothesis was that several procedural barriers to legal abortion prevent women from timely accessing abortion services in their area of residence and force them to seek abortion care outside their home departments.

We highlight the obstacles that women face in seeking abortion care in their department of residence. At the same time, we show that most of our respondents from Île de France commute to Paris for an abortion, not only to overcome existing obstacles to access, but also out of convenience.

In our research documents (information sheets, consent form), we used the term “women” to refer to participants, and we did not ask our participants to self-report their gender identity. However, we acknowledge that gender nonbinary and transgender people may also seek abortion. For the remainder of the article, when using the term women, we have an inclusive intent to denote women and pregnant people.

## 3. Methodology

This paper makes use of data collected during a five-year research project on barriers to legal abortion and abortion travel in Western Europe. It is based on a mixed-methods research methodology.

The study protocol was evaluated and approved by the ERC Ethics Committee on 4/03/2016 (ERCEA/BT/ ercea.b.1(2016)1090019). Ethical approval for this study was also granted by the University of Barcelona (Spain) on 13/02/2017, the University of Central Florida (US) on Feb. 21, 201 (SBE-17-12964), the University of Tillburg (Netherlands) on 23/03/2017 (EC-2017.22), the BPAS Research & Ethics Committee (UK) on 8/05/2017 (REC 2017/02/SDZ), the French Comité de Protection des Personnes on 24/10/2019 (ID-RCB: 2019-A01048-49), and the French Assistance Publique-Hopitaux de Paris on 02/02/2020 (APHP190995).

In this paper, we use (a) official abortion statistics, and (b) quantitative and qualitative data collected in three public hospitals in Paris, France.

(a) The statistical department of French Health Ministry (*Direction de la recherche*, *des études*, *de l’évaluation et des statistiques*, DREES) provided data of all abortions (*Interruption voluntaire de grossesse*, IVG), performed in hospitals and private clinics in France in 2019. We utilized data for metropolitan France only. Specifically, we analyzed and visualized variables containing information on the department of residence of and the department where the abortion took place for women seeking abortion within France. We computed abortion flows in 2019 (hospital and clinic) for metropolitan France. We used the open source cartographic software QGIS. We excluded travels for fewer than 20 abortions and showed results for metropolitan France and Île-de-France in separate maps to enhance the readability of the produced maps.

The maps were prepared using three steps. First, deleting the number 0 in the original Excel table, to search for data greater than or equal to 20 and to delete the data less than 20. Second, importing the "cleaned" data into QGis software (geographic information system). After and using the background of the French departments, the axes were entered (from the departments of residence to the departments where the abortions are performed). Five thresholds were applied to obtain the different flows (20–49; 50–99; 100–249; 250–499) for France. Two new thresholds were added for the Île-de-France region: 500–999; 1000–3818. The maps were exported in PDF. Third, we improved the readability of the maps using Adobe Illustrator software. The maps were exported in PNG and the layout was modified and adjusted.

(b) Quantitative and qualitative data were collected at three Parisian public hospitals between November 2019 and March 2020, and June and September 2020 by the leading author. Data collection was interrupted between mid-March 2020 and June 2020 due to the COVID-19 pandemic. Participating hospitals were selected based on available data about the annual volume of non-residents who obtain abortion care at the respective hospitals prior to the launch of the study. All hospitals had a very good reputation for their abortion services. These hospitals received also women who were close to the gestational age limit, but we could not recruit them due to ethical reasons and the organization of abortion provisions. Participants of this study were 18 years of age or older and were proficient in French, English, German or Spanish. Suitable individuals were identified by an on-site researcher and/or hospital staff and approached in a 2:1 ratio (2 travelers: 1 resident). Study participants were provided with an information sheet at the hospital. Those who expressed interest in participating could complete an anonymous, self-administered survey (tablet-based or paper-based), and/or take part in a confidential in-depth interview while they waited for their medical consultation prior to their abortion procedure. Safety protocols (including social distancing, wearing of masks, and disinfected tablets) were implemented during the pandemic. Informed written consent and oral consent were collected for all study participants. Participants were assigned a unique study identification number, and IDI participants could choose a pseudonym. All participant names in this paper are pseudonyms. The lead author translated the quotes from French into English.

Participants received a €10 gift card for surveys and a €25 gift card for interviews as compensation for their time. The survey included questions about women’s socio-demographic characteristics, reproductive history, pregnancy, abortion seeking experiences, experiences of inter-departmental travels, abortion stigma and experience with self-administered abortion. The interviews allowed for more in-depth exploration of participants’ experiences with the same range of topics covered in the survey, with a focus on barriers to abortion care in women’s department of residence and travel experiences.

Quantitative analysis was conducted using STATA software. Simple counts and percentages were calculated. We analyze variables descriptively and present responses broken down by participants’ origin (traveler vs. residents). Travelers are all those that reside outside Paris (department 75) or traveled longer than 60 minutes to the hospital. Residents are those residing in Paris (department 75). We acknowledge that in the Paris metropolitan area it is easy to commute via public transport and that some of the study participants residing outside department 75 (here classified as travelers) may have an actual travel time of under 60 minutes.

Qualitative analysis was conducted using Atlas.TI following a grounded theory approach. The interviews were coded using both predetermined codes initially created based on the main study themes, and new codes that emerged from the interviews. This article focused particularly on the most frequent code groups: access to abortions, barriers, and abortion information seeking.

We refer to the term “Planning Familial” which is the French family planning association and member of the International Planned Parenthood Federation. The association promotes sexual and reproductive health and rights and provides access to contraception, sex education and abortion consultation. Those Planning Familial centers with medical staff provide medical abortion up to 7 weeks of gestation. Otherwise, they provide abortion consultation and refer clients to abortion providers. Complementing the work of the Planning Familial associations, “Family Planning and Education Centers” (Centre de Planification et d’Éducation Familiale, CPEFs) are centers, often attached to hospitals, where health professionals, doctors, midwives, and counselors provide medical and surgical abortion care, among other services. In this paper, we use the term “Planning Familial” to refer to the association, and otherwise use family planning centers for the CPEFs.

## 4. Results

The results section is organized in three parts:

- Inter-departmental abortion travel in metropolitan France in 2019
- Quantitative findings
- Qualitative findings

### 4.1. Inter-departmental abortion travel, France, 2019

Data obtained from DREES offers insights into inter-departmental abortion travels in France.

These data show that overall 18% of abortions in France in 2019 were performed outside the women’s department of residence. This corresponds to abortion flows between women’s department of residence and the department of care provision.

**Fig 1:**
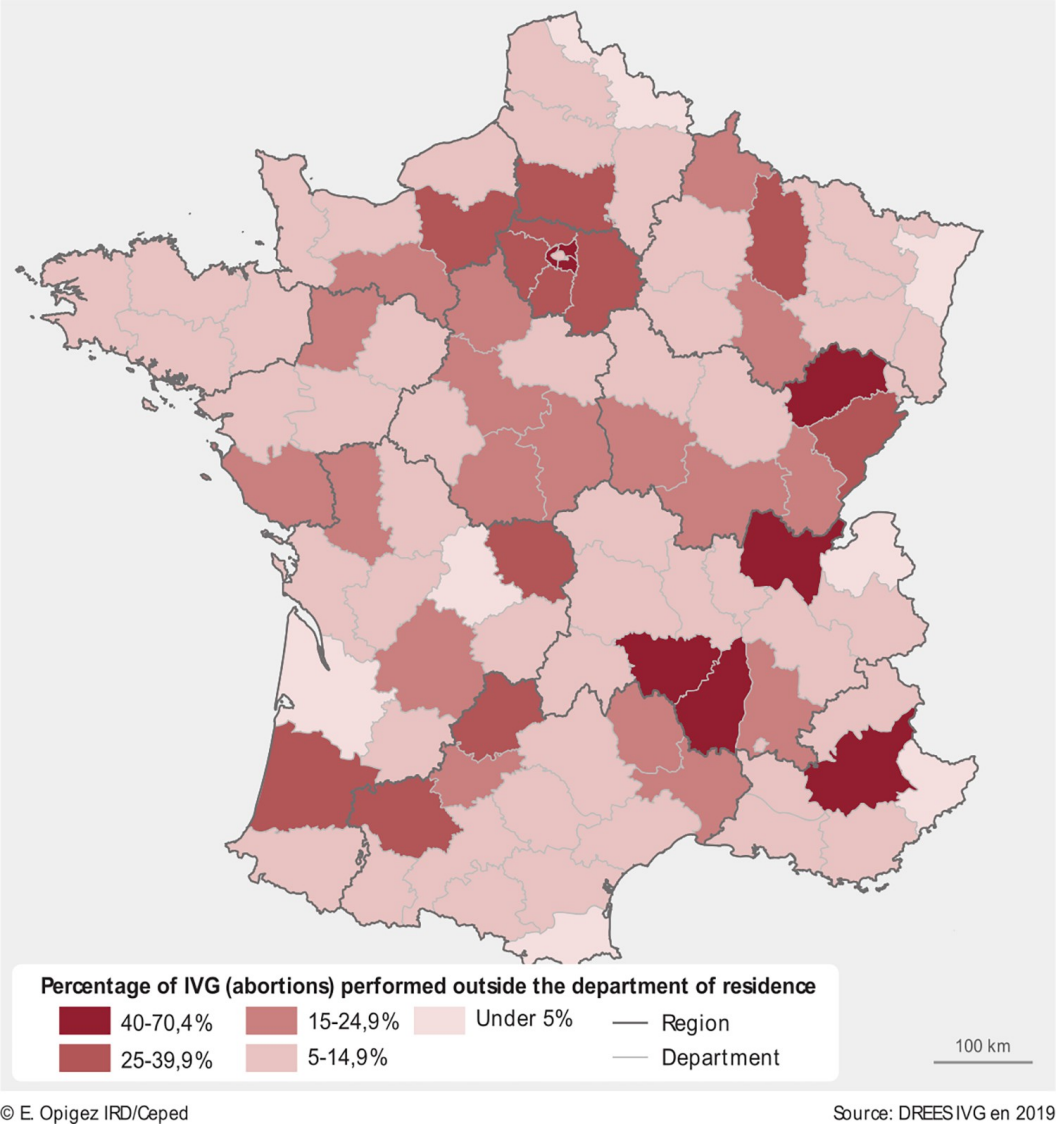
Percentage of abortions performed outside the department of residence, France, 2019. Source: [11]. Map produced by cartographer.

Fig 1 shows the percentage of abortions performed outside the department of residence in France in 2019. The highest concentration of abortions performed outside the department of residence (between 40–70%) can be observed a) in Île-de-France region (surrounding Paris, departments 92, 93, 94), and b) in the East (Haute Saone and Ain) and South-East (Haute Loire, Ardèche, Alpes de Haute Provence) of France. Topographically, the latter departments are in mountainous regions of France, the Massif Central, the Alps, and Jura mountains, with a relatively weaker infrastructure compared to the rest of France.

The second-highest concentration of between 25–40% of abortions performed outside the home department is among women residing in the Southwest (departments of Landes, Gers, Lot, Creuse), East (Doubs), and again in the greater Paris metropolitan area (departments 77, 78, 91, 95) as well as Oise and Eure, with smaller clusters (of 5–25%) being scattered across all French regions (Fig 1). Where do women travel to seek an abortion?

**Fig 2:**
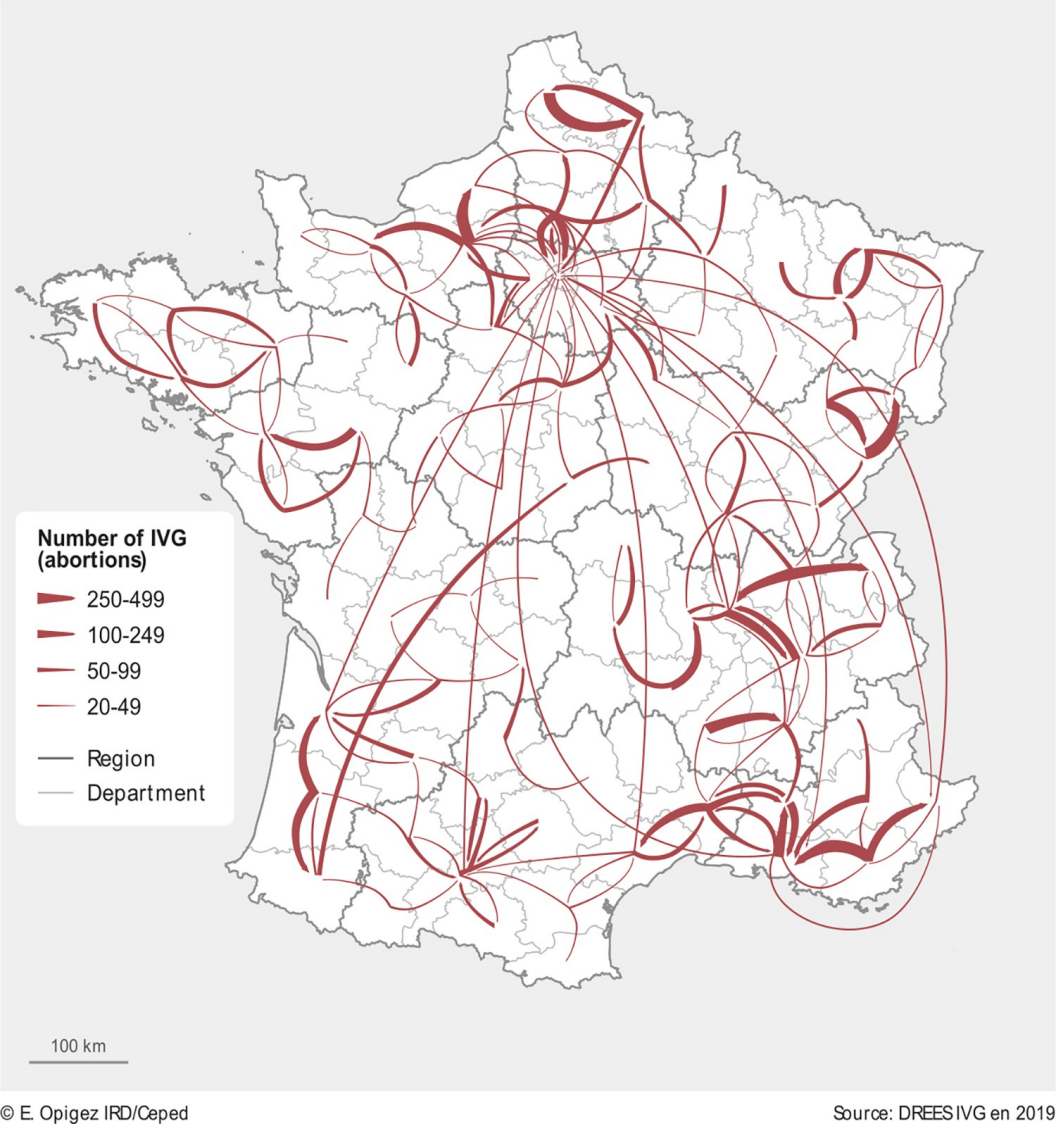
Inter-departmental abortion travels, metropolitan France, 2019. Source: [11]. Map produced by cartographer.

Fig 2 shows inter-departmental abortions travels in metropolitan France in 2019. The arrows indicate the trajectory from women’s department of residence to the departments where they received care. The bold arrows indicate the number of abortions with four thresholds (20–49; 50–99; 100–249; 250–499). Three dominant clusters for inter-departmental abortion travels become apparent, with cross-departmental travels exceeding 250 abortions in 2019 (Fig 2).

1. The Northern part of France concentrated around the regions Île-de-France, Haut-de-France and Normandy;
2. The Southeastern part of France concentrated around Auvergne-Rhône-Alpes and Province-Alpes-Cote D’Azur; and
3. The Eastern part of France around Grand-Est region.

In the Northern part of France, women from the department 27 (Eure) travel to surrounding departments, especially to 76 (Seine-Maritime). Similarly, there is mobility for abortion care from department 62 (Pas-de-Calais) to the neighboring departments, in particular 59 (Nord). Furthermore, women from department 60 (Oise) travel outside their department of residence, especially to Île-de-France region.

In the Southeast, we see major movement from department 01 (Ain) to department 69 (Rhône) and 74 (Haute-Savoie). Ain sticks out because 49% of women from Ain travel outside their department of residence for care. We also see mobility between department 69 (Rhône) and 38 (Isère) with almost an equal level of exchange (influx vs. outflow) between the two. Women also travel from department 43 (Haute-Loire) to 42 (Loire), and department 7 (Ardèche) to 26 (Drôme). The biggest fluctuations are visible along the coastline, particularly from 83 (Var) to 13 (Bouches-du-Rhône), and from 13 (Bouches-du- Rhône) to 84 (Vaucluse), and fewer trips from 30 (Gard) to 34 (Hérault) and 84 (Vaucluse).

In the northeast, abortion travels are concentrated in the departments bordering Switzerland and Germany. We see fluctuations in particular from 25 (Doubs) to 90 (Territoire de Belfort), as well as mobility between 54 (Meurthe-et-Moselle) and 57 (Moselle) and surrounding departments.

Smaller clusters of below 250 abortion travels can be identified on the margins of France: (i) mainly in the Southwest, where women are leaving department 40 (Landes) to obtain abortions in department 64 (Pyrénées Atlantiques) or 33 (Gironde); (ii) in the Bretagne region, where women are leaving department 22 (Côtes-d’Armor) to surrounding departments; and (iii) in Pays de la Loire region (with mobility between 85 (Vendée)), 49 (Maine-et-Loire) and 44 (Loire-Atlantique) (Fig 2). Fig 2 also shows that there is an overall mobility from various parts of France (e.g. departments 06, 13, 31, 33, 34 or 45) coming to Paris (75) for abortion care, yet in comparatively low numbers (below 49).

**Fig 3:**
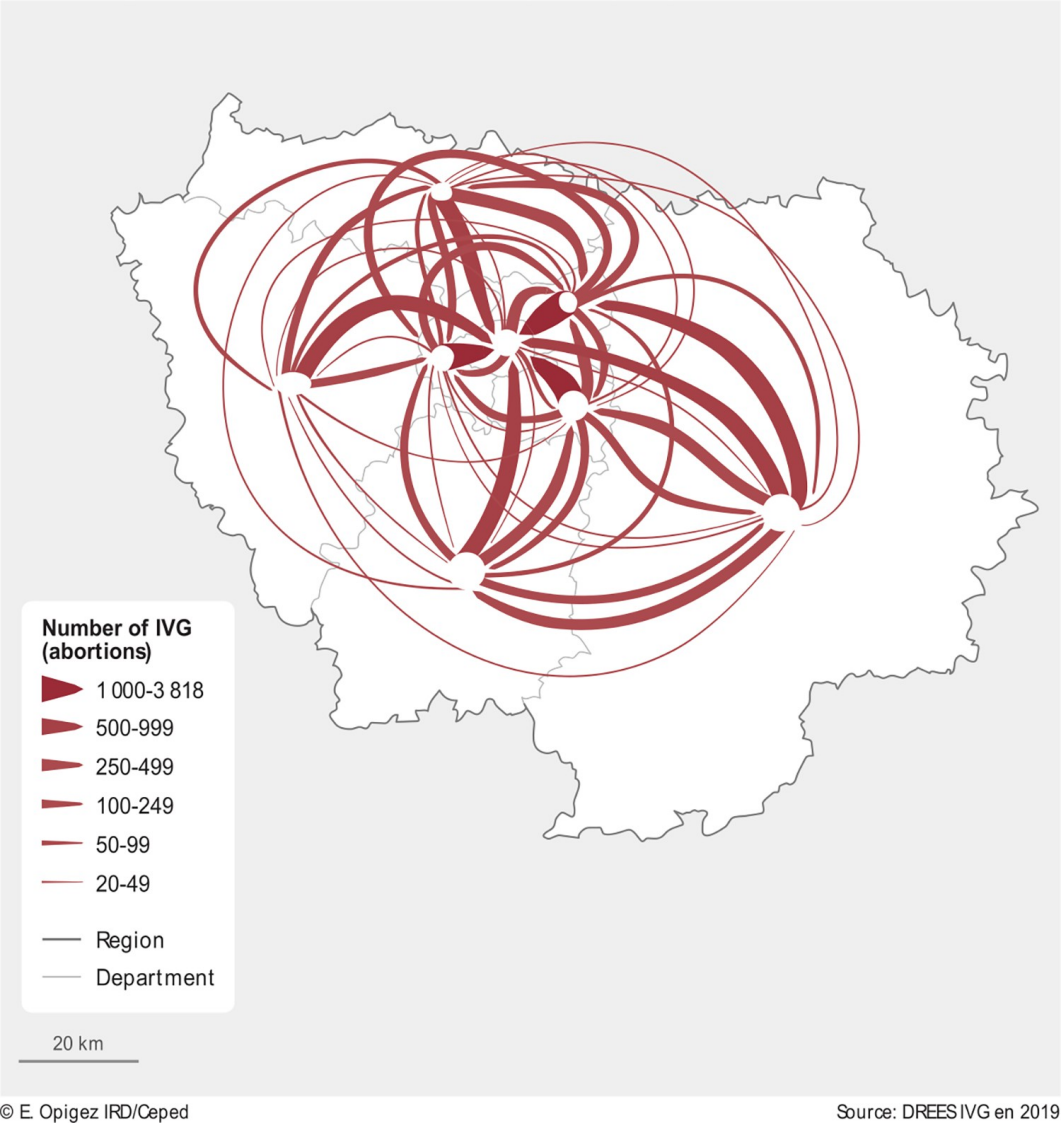
Abortion mobility, Île-de-France Region, 2019. Source: [11]. Map produced by cartographer.

Much higher mobility is evident within the Île-de-France region. Fig 3 shows inter-departmental abortion travels in Île-de-France in 2019.

Due to the high number of inter-regional mobility, the data are presented in a separate map (Fig 3) and excluded from Fig 1. We added additional thresholds (500–999; 1000–3818) to account for the high mobility in this region. Fig 3 shows that abortion travels in Île-de-France region are very frequent. We can identify three major trends in cross-departmental abortion flows.

1. *Coming to Paris*: The travelers come from the surrounding departments into Paris (75), especially from department 92 (Hauts-de-Seine, 3818 abortions), 93 (Seine-Saint-Denis, 3141 abortions), 94 (Val-de-Marne, 3027 abortions) with over 3000 abortion travelers in 2019, followed by 95 (Val-d’Oise, 924 abortions), 91 (Essonne, 851 abortions), 78 (Yvelines, 724 abortions), 77 (Seine-et-Marne, 678 abortions), and much less so from department 76 (Seine-Maritime, 35 abortions).
2. *Leaving Paris*: Women resident in Paris (75) are traveling outside their department, especially to department 93 (Seine-Saint-Denis, 359 abortions), 92 (Hauts-de-Seine, 167 abortions), and less so to 94 (Val-de-Marne, 88 abortions).
3. *Mobility around Paris*: Lastly, there is mobility around Paris. Department 92 (Hauts-de-Seine) receives around 610 women from surrounding departments 91, 93, 94, 95. Furthermore, women resident in 77 (Seine-et-Marne) travel to 91 (Essonne, 256 abortions) with almost an equal number traveling in the opposite direction from Essonne to Seine-et-Marne (244 abortions).

Additionally, smaller clusters (20–49; 50–99 abortions) can be identified throughout Île-de France (Fig 3). These maps show that inter-departmental abortion travels are national phenomenon in France, and that Île-de-France region accounts for the greatest share of inter-departmental travels in 2019.

### 4.2. Quantitative findings

Drawing from 172 surveys with women seeking abortion services in Paris, 100 participants traveled outside their department of residence within France to receive services in Paris and 72 participants were residents of Paris. Most of the 100 travelers reside in Île-de-France. Only 6 of them came from outside Île-de-France, including Auvergne-Rhône-Alpes, Centre-Val de Loire, Corse, Hauts-de-France, Normandy and Pays de la Loire. Of these, 5 were temporarily based in or regularly traveled to Île-de-France for work or study-related reasons. We examine the socio-demographic characteristics of women traveling to seek abortion care in Paris in contrast to resident women, their reproductive history and experiences in seeking abortion care, the reasons for travels, travel experiences and costs in accessing abortion services, as well as more broadly barriers to legal abortion.

#### 4.2.1. Socio-demographic characteristics

Table 2 provides the demographic profile of survey participants. Over one third of participants were 18 to 24 years old (38%), the same share (38%) was aged 25–34, and the remaining 24% were 35 years or older. Travelers were noticeably younger than resident women: almost half of all travelers were aged 18–24 (48%) vs. only 24% of resident women were in this age group. Meanwhile, 35% of residents were 35 years or older, while only 16% of travelers belonged to this age group.

**Table 2:** Socio-demographic characteristics.

	Travelers (n = 100)	Residents (n = 72)	All (n = 172)
**Age**			
18–24	48 (48%)	17 (24%)	65 (38%)
25–34	36 (36%)	30 (42%)	66 (38%)
35–49	16 (16%)	25 (35%)	41 (24%)
**Highest level of education completed**			
Secondary school or below	15 (15%)	12 (17%)	27 (16%)
Some university	21 (21%)	12 (17%)	33 (19%)
University or graduate school	41 (40%)	27 (38%)	68 (39%)
Post-graduate	19 (19%)	19 (26%)	38 (22%)
Prefer not to answer/no response	6 (6%)	2 (3%)	8 (5%)
**Employment** ^**a**^			
Employed full-time	45 (45%)	32 (44%)	77 (45%)
Employed part-time	15 (15%)	8 (11%)	23 (13%)
Self-employed	6 (6%)	11 (15%)	17 (10%)
Unemployed	16 (16%)	5 (7%)	21 (12%)
Student	20 (20%)	14 (19%)	34 (20%)
Other	3 (3%)	3 (4%)	6 (3%)
**Ability to meet basic needs**			
All or most of the time	62 (62%)	60 (83%)	122 (71%)
Some of the time	16 (16%)	4 (6%)	20 (12%)
Never or rarely	15 (15%)	5 (7%)	20 (12%)
Prefer not to answer/no response	7 (7%)	3 (4%)	10 (6%)
**Marital status**			
Married or in a civil partnership	33 (33%)	32 (44%)	65 (38%)
Single, separated, or divorced	61 (61%)	35 (49%)	96 (56%)
Other	4 (4%)	4 (6%)	8 (5%)
Prefer not to answer/no response	2 (2%)	1 (1%)	3 (2%)
**Religious Affiliation**			
Catholic	26 (26%)	10 (14%)	36 (21%)
Protestant	3 (3%)	3 (4%)	6 (3%)
Muslim	19 (19%)	14 (19%)	33 (19%)
Atheist/Agnostic	32 (32%)	39 (54%)	71 (41%)
Other	8 (8%)	1 (1%)	9 (5%)
Prefer not to answer/no response	12 (12%)	5 (7%)	17 (10%)

^a^ More than one answer was possible, percentages may exceed 100%.

Source: data collected and compiled by authors.

The majority of participants had attended or completed university (58%). Slightly fewer travelers than resident women held a postgraduate degree (19% vs. 26%). Most respondents were employed in some capacity (68%), and had sufficient resources to meet their basic needs (83%). A higher proportion of travelers reported being unemployed compared to residents (16% vs. 7%) and having insufficient income to meet their basic needs (15% vs. 7%).

Over half of all respondents were either single, or separated, or divorced (56%), with slightly more travelers than residents (61% vs. 49%). 38% respondents reported being married or in a civil partnership. We also observed some descriptive differences between the religious backgrounds of participants. One third of travelers (32%) identified as Atheist or Agnostic, while over half of resident women (54%) did not follow any religion. Travelers identified more often than residents as Catholic (26% vs. 14%) (Table 2).

### 4.2.2. Reproductive history and experiences in seeking abortion care

Table 3 provides information about participants’ reproductive histories, and experiences seeking abortion care in their department of residence. About two thirds of participants had no children (66%) and had never had a prior abortion (57%).

**Table 3:** Reproductive history and abortion care-seeking.

	Travelers (n = 100)	Residents (n = 72)	All (n = 172)
**Number of children**			
0	66 (66%)	48 (67%)	114 (66%)
1–2	23 (23%)	18 (25%)	41 (24%)
3+	0	6 (8%)	6 (3%)
Prefer not to answer/no response	11 (11%)	0	11 (6%)
**Prior abortion**			
Yes	34 (34%)	25 (35%)	59 (34%)
No	55 (55%)	43 (60%)	98 (57%)
Prefer not to answer/no response	11 (11%)	4 (6%)	15 (9%)
**Weeks of gestation when presenting for services**			
1–6 weeks	49 (49%)	36 (50%)	85 (49%)
7–12 weeks	48 (48%)	30 (42%)	78 (45%)
13 weeks	2 (2%)	1 (1%)	3 (2%)
Prefer not to answer/no response	1 (1%)	5 (7%)	6 (4%)
**Mean weeks of gestation when presenting for services**	6.7	6.4	6.6
**First considered abortion before presenting for care**			
Less than 2 weeks	36 (36%)	29 (40%)	65 (38%)
2–3 weeks	19 (19%)	18 (25%)	37 (22%)
3–4 weeks	14 (14%)	10 (14%)	24 (14%)
4 weeks or more	14 (14%)	10 (14%)	24 (14%)
Prefer not to answer/no response	17 (17%)	5 (7%)	22 (13%)
**Sought abortion elsewhere before presenting for care in hospital in Paris**			
Yes	31 (31%)	6 (8%)	37 (22%)
No	69 (69%)	65 (90%)	134 (78%)
Prefer not to answer/no response	0	1 (1%)	1 (1%)
**Preferred to obtain abortion earlier**			
Yes	73 (73%)	42 (58%)	115 (67%)
No	24 (24%)	28 (39%)	52 (30%)
Prefer not to answer/no response	3 (3%)	2 (3%)	5 (3%)
**Reasons for not being able to obtain an abortion as early as wanted** ^**b**^			
No delays/Obtained abortion when wanted	24 (24%)	28 (39%)	52 (30%)
Delayed pregnancy recognition	32 (32%)	15 (21%)	47 (27%)
Issues with scheduling (both personal and getting an appointment at the clinic)	27 (27%)	17 (24%)	44 (26%)
Procedural barriers including waiting periods, need for multiple approvals, or attending multiple appointments	21 (21%)	17 (24%)	38 (22%)
Delays related to local access to abortion services	25 (25%)	10 (14%)	35 (20%)
Delays related to decision-making	22 (22%)	9 (13%)	31 (18%)
Delays related to a change in the situation (financial, relationship, decision-making)	13 (13%)	4 (6%)	17 (10%)
Difficulties arranging money for abortion	1 (1%)	1 (1%)	2 (1%)
Issues arranging travel	1 (1%)	1 (1%)	2 (1%)
Others	4 (4%)	1 (1%)	5 (3%)

^a^ More than one answer was possible, percentages may exceed 100%.

Source: data collected and compiled by authors.

Half of the respondents were 1–6 weeks pregnant (49%) and the other half were 7–13 weeks (47%). Thirty-eight percent of participants first considered abortion less than two weeks before presenting for care in Paris. Meanwhile, under one third of participants (28%) considered an abortion more than four weeks before presenting for care. There were no notable differences between traveling and resident women concerning these variables. Respondents were asked whether they sought abortion elsewhere before coming to the hospital in Paris, where they were recruited. One third of travelers searched for abortion care elsewhere before traveling to Paris (31%) while only 8% of resident women searched for care elsewhere. Travelers also reported more often than residents that they would have preferred to obtain an abortion earlier in their pregnancy (73% vs. 58%), even though we did not observe differences in the gestational age between residents and travelers (Table 3).

The main reasons why respondents reported not obtaining care earlier were: delayed pregnancy recognition, scheduling, procedural barriers, a lack of local services, decision-making factors, and change in personal circumstances. Many participants cited more than one reason. Compared to residents, a higher proportion of travelers reported delayed pregnancy recognition (32% vs. 21%). The differences in delays cited more frequently by travelers were due to local access to services (25% vs. 14%), decision-making (22% vs. 13%), and a change in circumstances (13% vs. 6%). 34% of travelers had had a prior abortion. Some of them reported their past negative experiences as one of the reasons why they had decided to go to Paris for abortion care. Arranging for travels was not a factor that delayed women’s access to care. Most of the 100 traveling participants recruited in Paris come to the French capital on a regular basis: 57% several times a week (2–7 times), mainly for work, studies, medical care, shopping, or leisure. Only 20% do not come to Paris in an average month. For most, traveling to Paris for abortion care represents another facet of their usual commuting experience.

#### 4.2.3. Reasons for travels

As the primary reason for seeking abortion care in Paris, respondents indicated (i) a referral by a healthcare provider (21%), (ii) concerns about the quality of abortion care in their department of residence (20%), or (iii) lack of knowledge about where to get an abortion in their own department (12%). For 3–7% of respondents the main reasons were: prior knowledge of the hospital, timely appointments, proximity, and the hospital’s reputation. Other reasons were linked to privacy, preferred abortion method, and anticipated judgment or refusal. When asked why women chose one specific hospital over another, the answers were somewhat similar: almost half of respondents (46%) were referred to the specific hospital either by a healthcare provider, friend or relative. Meanwhile, close to one third of respondents named the good reputation (31%) or easy access of the hospital (27%) as reasons for their trips (Table 4).

**Table 4:** Reasons for traveling for abortion care.

	Travelers (n = 100)
**Primary reason for traveling**	
A health provider referred me	21 (21%)
I was concerned about the quality of abortion in my department	20 (20%)
I didn’t know where to get an abortion in my department	12 (12%)
I already knew the hospital	7 (7%)
The hospital had the earliest available appointment	6 (6%)
I was worried that people I know would see me at the clinic	5 (5%)
It is difficult to find a physician in my department who is willing to provide care	5 (5%)
Close proximity	4 (4%)
The reputation of the hospital	3 (3%)
I was worried about someone finding out about my abortion	3 (3%)
I wanted to have a medical termination, which was not available at my department	3 (3%)
I could not obtain an abortion at my gestational age in my department	3 (3%)
There are no abortion services where I live	2 (2%)
I was worried about being judged by healthcare providers in my department	2 (2%)
A friend or family member referred me	2 (2%)
I wanted to have a surgical termination, which is not available in my department	1 (1%)
I was worried about a healthcare provider refusing to provide care in my dept.	1 (1%)
**Reasons for traveling to specific hospital over another in the department**	
I was referred by the health-care providers in my department	33 (33%)
It has a good reputation	31 (31%)
It was the easiest to get to	26 (27%)
I was referred by someone else (doctor, friend, family)	13 (13%)
It was the easiest to find online because it pops up first or the most often	7 (7%)
It was the closest one which provides abortion at my gestational age	4 (4%)
The hospital had the earliest available appointment	4 (4%)
The cost of traveling to this hospital was the cheapest or the abortion was cheapest	3 (3%)
Other	1 (1%)

^a^ More than one answer was possible, percentages may exceed 100%.

Source: data collected and compiled by authors.

#### 4.2.4. Travel experiences and costs

Regarding participants’ travel experiences and costs, most participants (71%) used public transport to come to the hospital. Travelers used a personal car more often than residents (31% vs. 11%). Overall travel costs were relatively low, for both travelers and resident women, with 91% of the respondents spending less than €10, and 89% reporting that it was very or somewhat easy to cover travel costs. Half of the respondents (49%) took time off work for the abortion consultation, with fewer non-resident (44%) than resident women (57%). Conversely, more non-residents than residents reported to have lost wages due to abortion care seeking (34% vs. 22%). Only 13% of respondents had to arrange for childcare while seeking abortion, with fewer travelers than residents (8% vs. 19% respectively).

### 4.3. Qualitative findings

Qualitative in-depth interviews were conducted with 39 women in three Parisian hospitals (23 travelers and 16 residents). These provide further insights into the diverse reasons why participants in our study traveled to Paris to seek abortion care. Focusing on 23 travelers, we discuss the major themes that arose from the qualitative analysis with regards to women’s barriers to abortion care in their department of residence and their reasons for traveling. These are, in order of relevance: 1) referral by healthcare providers, 2) long waiting times in the department of residence, and 3) easy access to quality care in Paris. Most women reported several of these reasons in combination. Each theme is illustrated with quotes that are representative of the overall responses. The analysis broadens our understanding of inter-departmental abortion travel and the experiences of women undertaking such travels.

#### 4.3.1. Referral by healthcare providers: Diverse push and pull factors

Confirming the quantitative data, the main reason for traveling to Paris was the referral by a healthcare provider in the department of residence. 19 out of 23 respondents stated that health professionals (mainly gynecologists, general practitioners, local Planning Familial centers, and midwifes) but sometimes also a friend or relative referred them to Paris or to the specific hospital. Referrals were associated with diverse push and pull factors, including (a) the lack of services in the department of residence, and (b) a good reputation of the Parisian hospitals providing care:

(a) Six interview participants stated that services were lacking in their department of residence. Diane, 40 years old, who was 9 weeks pregnant when we met her, had traveled from Essonne, where she was told that there were no abortion services close to where she lived. She stated, *“I went to my general practitioner first*, *who told me that at the hospital in my town*, *they don’t do abortions*, *at all… So*, *she told me to go to the Planning Familial center and that they would refer me”* (interviewed 10.3.2021). Diane was in fact referred several times: her general practitioner provided the Planning Familial center contact; the center referred her to several hospitals in Paris; and lastly Diane was also advised by close friends to contact the specific hospital where she finally received care. Other respondents reported similar cases. Clemence, 49 years from Val-d’Oise, first called hospitals in her area but never got any response. When asked why she traveled to Paris, Clemence stated, “*simply because in my department I found that abortion was not so easy to find*. *The hospitals I contacted*, *well*, *they never answered me*” (interviewed 24.6.2020). Finally, her adult daughter, who also had an abortion, referred her to the specific hospital, where she would receive quality care.

(b) Nine interviewees reported that they approached the Planning Familial for abortion consultations, and usually received several options of providers. In Diane’s case above, her friends helped her choose the hospital among the list of Paris-based providers. In other cases, the Planning Familial staff supported the women in finding a hospital, based on the hospital’s reputation. The case of Anna, 26 years old, 5 weeks pregnant and originally from Centre-Val-de-Loire Region, 160 km South of Paris, provides an example. At the time of the interview, Anna was unemployed and in Paris for a month-long professional training. When she learned she was pregnant, Anna did not want to travel back home for an abortion as in her small city there are fewer abortion centers than in Paris. Anna obtained a list of providers from a Parisian Planning Familial. She stated, *“And we looked at the closest one*, *actually*, *to where I’m staying*. *So there were two*. *And she [Planning Familial staff] recommended the one where I am now*, *because she told me that she knew the doctor and that she was very good*. *So it’s true that I went to this one”* (interviewed 9.3.2021). The referral was linked to the good reputation of the doctors providing care and the proximity of the hospital to her temporary lodging in Paris (see theme 3 below).

Confirming the quantitative findings, travelers regularly came to Paris for work or medical treatments. This was Lea’s case, 32 years old, 3 weeks pregnant, and resident in Yvelines (78), who commuted to Paris for work. She reported that it was easier for her to seek medical services in Paris since she lived in a rural area. She stated, “*My gynecologist is in Paris*. *Since I am often in Paris*, *it is more convenient for me*, *because I live in the countryside and there are not many of them [gynecologists]*. *And my gynecologist*, *she also works at this hospital… it’s thanks to her that I was accompanied at 100%*” (interviewed 15.3.2021). The quote highlights the interconnection of referral with lack of services in the department of residence and good reputation of quality care in the Parisian hospital. While the lack of services pushes women to seek care outside their region, there is also a strong pull factor to come to Paris to receive quality abortion care in a timely manner.

#### 4.3.2. Long waiting times causing delays in abortion seeking: A stressful experience

The second theme that emerged from the qualitative data was slow access to care as a major reason for inter-departmental abortion travel. 13 non-residents reported that they faced long waiting times in their department of residence, and many were offered appointments only after 1 or 2 months, meaning they would have been close to or beyond the legal gestational age limit. Julie, a 22 years old, 7 weeks pregnant woman who traveled from Haute-Normandie, reported,

*“I looked for [abortion care in my department]*… *Well*… *at the hospital*, *they said it was going to take too long*, *and I couldn’t wait too long because to do an abortion there are dates*, *deadlines*. *Except that there were no appointments …*, *so I looked elsewhere*, *and I found it… I searched online and I was offered a place here*, *and since I work in Paris*, *I said*, *I’m going to come here at once"* (interviewed 6.5.2020).

Julie received care at 7 weeks gestation, and since she works in Paris it was easy for her to undergo treatment there. Had she waited for an appointment close to her residence, she would have exceeded the legal gestational age limit for receiving an abortion in France. Several other travelers reported similar scenarios, often coming from surrounding departments. Clemence, aged 24 from Seine-Saint-Denis (93), 7 weeks and 3 days pregnant, stated:

*“As soon as I found out I was pregnant*, *at one or two weeks*, *until today*, *I couldn’t find a doctor*. *I couldn’t get an appointment*. *I couldn’t get anyone on the phone*. *It was complicated*. *And the only clinics I saw told me always the same*: *by medication*, *medication*. *Whereas here*, *it was very quick*, *they accepted my choice and gave me an appointment as soon as possible”* (Clemence, interviewed on 24.6.2020).

Clemence reported that it was a very “*stressful*” and “*agonizing*” period because getting an appointment in her area was so complicated. It took her 6 weeks to finally find a hospital. The available appointments in Paris, on the contrary, were much more favorable and faster. For Clemence, getting an appointment was not the only concern. Surgical abortions—her preferred procedure—was not offered in her department of residence. Five other respondents stated similar constraints in the lack of the preferred abortion procedure in their department of residence. Coming to Paris was hence seen as a strategy to avoid long waiting periods, receive the desired abortion procedure, and ensure access to timely care.

#### 4.3.3. Easy access to quality care

The third theme that arose from the qualitative data as a major reason for inter-departmental abortion travel was easy access to quality abortion care in Paris. Eight participants reported that they went to Paris because high quality services were easily accessible with short commuting distances. It needs to be stressed that 94% of the non-residents come from Île-de-France region with good transport connections to the capital city.

Maeva, 37 years old, 3 weeks pregnant, from Seine-Saint-Denis (93) looked online for a Planning Familial center. She reported, “*I searched on the Doctissimo website to know where I could find the nearest Planning Familial center*. *I preferred to go to a Planning Familial rather than a gynecologist [in town]*, *and this was the closest one to my house*” (interviewed 13.3.2021). She ended up traveling 10 minutes via Uber to the hospital.

Similarly, Cecile, 28 years old, reported that coming to Paris was easy for her as it is within close reach. She stated, “*I live in the Paris region*, *in the 94th department*, *and I am very close to Paris*. *So*, *another city in my department or Paris*, *it’s the same thing*. *In terms of distance*, *it’s the same*. *For me*, *what was important was to know that I knew someone who knew these professionals and that it went well for her*, *to reassure me that it was going to go well for me too*” (interviewed 9.3.2021). For Cecile, both the proximity to services and a trusted source of referral were important to assure her as to the quality of care. In Cecile case, it was a friend who had an abortion at the specific hospital before and who encouraged her to see the same doctors (also see theme 1).

We can see that theme 3 is interconnected with theme 1. The following quote by Diane, cited earlier, is paradigmatic of many voices from the field: “*I was advised to go to [name of hospital] because it was convenient*, *there was good care*, *and I didn’t mind driving a few miles*, *since I have a car*. *It wasn’t a problem for me*” (interviewed 10.3.2021). Diane had difficulties in finding services in her department of residence, the hospital in Paris was recommended due to its good reputation and was easy and convenient to access by car.

Even respondents who do not own a car reported that coming to Paris via public transport was easy, and sometimes even quicker than seeking services in their own department. For example, Jeanne, 26 years old and 7 weeks pregnant, traveling from Val-d’Oise, stated, “*it’s true that with public transportation*, *it’s often easier to get to Paris than to be in the suburbs*. *Sometimes it’s faster*.” Jeanne continued by stressing that, “*in terms of transportation … and mileage*, *I’m not very far*. *And I had greater offers in Paris*. *But anyway*,… *I would have preferred Paris*, *because*, *it’s a big city*, *and maybe more open-minded than where I am”* (interviewed 15.3.2021). Jeanne, as most other respondents, was referred to Paris (see theme 1), because she could get easy access to quality care, which she was unavailable in her area of residence.

The six respondents who reside outside Île-de-France reported about limited access to abortion services in their department of residence and frequent travels to Paris for work, study, or leisure. Two of them expressed privacy concerns as a motivation of their travels. This was the case of Valentine, 23 years, 6 weeks pregnant, African descent and resident of Rhône-Alpes, 500 km Southeast of Paris. When being asked about the reason of her travel, she reported, “*Because I don’t want to do this in my city*. *I know a lot of people in my city that might judge me*” (interviewed 24.1.2020). The fear of being exposed and humiliated (“*In my city people … might judge me and laugh at me like*: *“Ha ha*, *you have done an abortion*.*”*) pushed her into traveling 8 hours via Flix Bus to the French capital (“*I just had to escape from my city and come to Paris*”). This interview was the exception in terms of travel time, as most of our interviewees commuted to Paris from within Île-de-France.

## 5. Discussion

The findings contribute to an emerging body of literature demonstrating that women living in countries with relatively liberal abortion laws nevertheless undertake travels for abortion care. A systematic review [20] synthesized the existing literature on abortion travel, the majority of which focused on travel within the United States—a country with a state-by-state legal patchwork of abortion access—and focused on understanding and quantifying the burdens related to travel, and documenting the impact travel experiences on outcomes such as desired abortion method. Our study adds to the existing literature on inter-departmental abortion travel by offering novel insights into the experiences of women who do not find services in their home departments, in a country where abortion has been legal on broad grounds for decades.

Based on abortions data in France in 2019 and maps tracking inter-departmental abortion travels, our study shows that the highest number of abortion travels in 2019 took place within the Île-de-France region.

Drawing on our quantitative findings and the descriptive analysis of the socio-demographic characteristics, our study revealed that traveling women are slightly younger and more economically vulnerable than resident women. These differences may be due to the different socio-demographics of the population living in the city of Paris and in the rest of Île de France. Regarding their reproductive history and abortion care-seeking in the department of residence, travelers and resident women showed a similar profile, with the exception that over 30% of travelers searched for care elsewhere before coming to the hospital where they received care, while this applied only 8% for resident women. This suggests that traveling women had an expectation they could access care locally. More travelers than residents preferred to have an abortion earlier and reported delays related to local access to abortion services. Our survey data show that travelers, more often than residents, face barriers to accessing abortion care where they live, but are also delayed by decision-making and changes in their personal situation (Table 3).

Primary reasons for abortion travels to Paris were (i) referral by a healthcare provider, (ii) concern about quality in the department of origin, and (iii) easy access to hospital in Paris. In terms of travel experiences and cost, for most travelers going to Paris did not represent a financial challenge. Non-residents who are more economically vulnerable than resident women took less time off work, compared to resident women. However, travelers, due to their more vulnerable employment status, lost more wages compared to resident women, highlighting the need for improved access to abortion care for women living outside of metropolitan areas.

The qualitative analysis complemented the quantitative data. It provided further insights into barriers to legal abortion and motivations of women undertaking inter-departmental abortion travels: respondents were strategically referred to come to Paris, where they can easily access quality services, which they lack in their department of residence. Several factors, in particular long waiting times and lacking services in the department of residence, push women into such travels. Meanwhile, different pull factors, such as the referral to high quality abortion services, motivate women to undertake such trips. For the most parts, the qualitative findings resembled the quantitative data. However, we found that, while 20% of survey respondents had concerns about the quality of abortion care in their department of residence, similar concerns were not expressed in the qualitative interviews. Only one of the 23 interview participants was concerned about the quality of care in the department of residence, through indirect experiences of a distant friend. Interview participants rather commented on the quality of care they could receive by traveling to Paris. Overall, women who participated in our study did not necessarily perceive their traveling as a problem, since it was relatively easy and inexpensive. Many expressed that it was a matter of choice because coming to Paris allowed them quick, easy access to high-quality care. Commuting was seen as a practical solution to overcome barriers to accessing abortion care locally.

## 6. Conclusion

18% of women in France travel outside their department of residence to receive abortion care (S1 Table). Various barriers exist for why women do not find services (lack of services or access to methods, quality of care, long waiting times). Inter-departmental abortion travels in France are national phenomena. However, most travels take place within Île-de-France region. Interviewees (S2 Table) report coming to Paris as a convenience and use travels as a strategy to overcoming obstacles in receiving care.

Despite governmental efforts in improving access to abortion care throughout the country, our study’s results show that women frequently do not find timely and quality services in their department of residence and hence travel for abortion care. The vast majority of those who traveled to Paris would have preferred to access abortions earlier, and 25% of them were not able to find services locally. One in three non-residents looked for services before coming to the hospital in Paris (versus 8% or residents), and could not find one close to their home. Our results highlight the stratification of access to abortion care, which is more difficult in non-urban areas and small urban centers than in big cities, like Paris, particularly for younger women with limited financial resources. Procedural barriers that can delay access to abortion, particularly long waiting periods, should be removed to allow women to obtain necessary healthcare in their own communities without impediments, and thus ensure their fundamental reproductive rights.

## 7. Strength and limitations

This study provides new data on a poorly understood phenomenon, inter-departmental abortion travels in France. The strength of the paper is to offer a robust understanding of the Île-de-France regional travel for abortion care. However, we only recruited study participants in Paris, the majority of whom come from Île-de-France. We acknowledge distinct socio-demographic backgrounds (particular between travelers and residents) that are not representative of all of France. We would have liked to (a) recruit more travelers from outside Île-de-France and (b) collect data in other major abortion travel destinations, but were unable to do so because of mobility restrictions in the course of the COVID-19 pandemic. We would also have liked to include in our sample participants who had traveled to Paris to obtain an abortion close to the GA limit, but could not do that due to ethics concerns and the organization of abortion services, as explained in the introduction. Finally, we did not include in our study private abortion services and may thus have missed some of the people who traveled to Paris from other departments and regions.

## Supporting information

S1 Table Abortion mobility metropolitan France in 2019. Department of women’s residence (in rows) by department of abortion realisation (in columns). Mobilities under 20 abortions were deleted.
(XLSX)

S2 Table Profile of abortion travelers participating in in-depth interviews. (XLSX)
